# Successful repair of recurrent ventricular septal perforation after myocardial infarction using double patch technique via right ventriculotomy: a case report

**DOI:** 10.1186/s13019-024-02673-3

**Published:** 2024-04-20

**Authors:** Khoirur Rijal Ashsholih, Taiichi Takasaki, Mayu Tomota, Taika Tokumoto, Seimei Go, Shogo Emura, Masamichi Ozawa, Shinya Takahashi

**Affiliations:** 1 Department of Surgery, Graduate School of Biomedical & Health Sciences, Hiroshima University, 1-2-3 Kasumi, Minami-ku, Hiroshima, Hiroshima 734-8553 Japan; 2 Department of Cardiovascular Surgery, Hiroshima University Hospital, Kasumi 1-2-3, Minami-ku, Hiroshima, Hiroshima Japan

**Keywords:** VSP after MI, Recurrent VSP, Double patch technique

## Abstract

**Background:**

Post-myocardial infarction (MI) ventricular septal perforation (VSP) is a rare but life-threatening complication. Surgical repair is challenging and carries significant risks, particularly in the context of recurrent VSPs. This case study presents a patient with recurrent VSP after initial surgical repair following myocardial infarction.

**Case presentation:**

A 65-year-old male were re-administered to our hospital due to recurrent VSP. He was during follow up after undergone emergency VSP closure surgery 2 months earlier, utilizing the bovine double patch technique via left ventriculostomy. The initial VSP was located in the apical part of the interventricular septum, while the recurrent VSP appeared in the upper middle portion of the interventricular septum (Fig. 1). As the previous patch remained intact, the second surgery employed the bovine double patch technique via right ventriculostomy. The patient’s condition remained stable without the development of heart failure symptoms.

**Conclusion:**

Repairing recurrent VSPs remains a challenge, necessitating the mastery of appropriate approaches to achieve optimal outcomes. Further research and guidelines are required to refine management strategies for recurrent VSPs.

## Background

The occurrence of a post-myocardial infarction ventricular septal perforation (VSP) is an infrequent but often fatal complication, affecting less than 1% of patients who have experienced myocardial infarction (MI) in the modern era of early reperfusion therapy [1]. Mortality rates among VSP patients after MI range from 19 to 60%, even with surgical interventions [2]. Repairing a recurrent VSP can be highly challenging and carries an additional mortality risk of 13–31% [3].

**Fig. 1:**
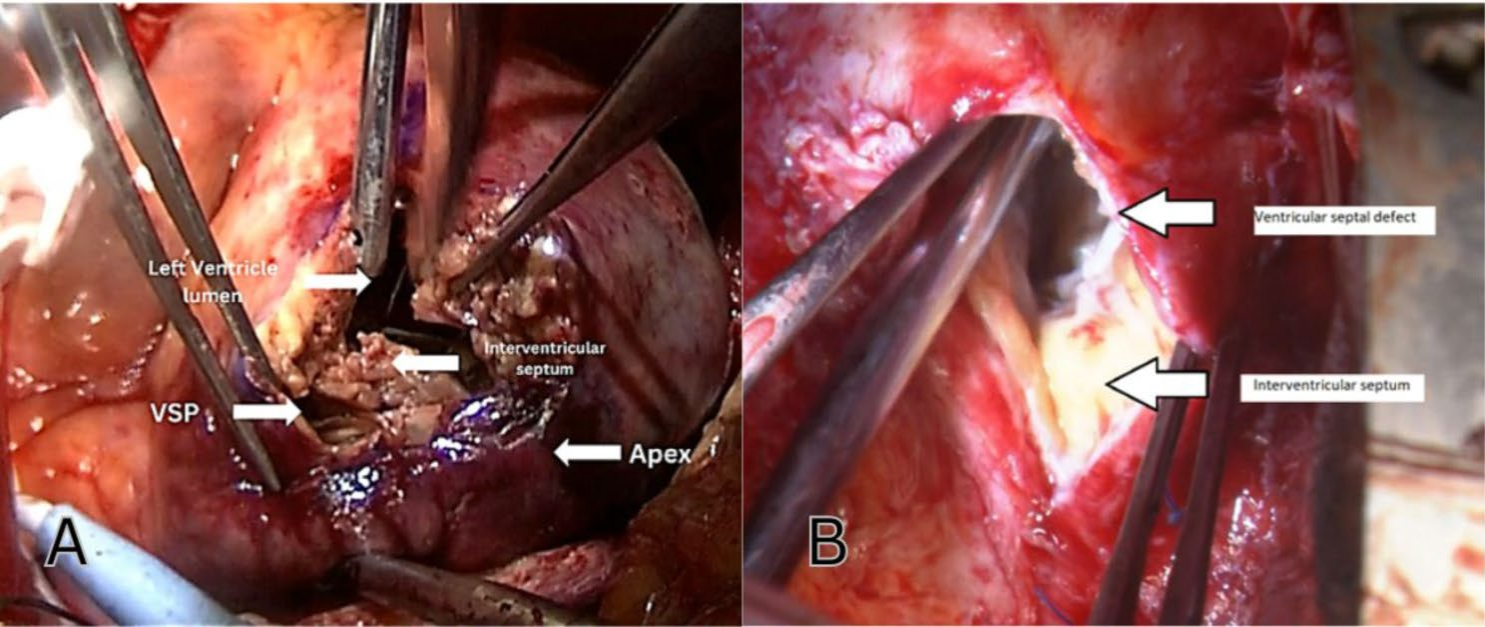
(**A**) Image of the first surgery, the VSP located near the apex. (**B**) Image of the second surgery, the VSP located in the upper middle of the interventricular septum

Despite the relatively high mortality rates and complex nature of the condition, surgical repair remains the best treatment option for VSPs following MI [4]. Prompt surgical intervention after a myocardial infarction offers the most favorable outcome for patients with post-MI VSPs. However, VSP recurrence occurs in approximately 5–11% of cases [5]. Recurrence is often due to infarct extension, jeopardizing initial surgical repair and leading to patch detachment or new VSP emergence [3, 5]. In this article, we present a case of new VSP formation after successfully closing a previous VSP following MI (See Fig. 1).

## Case presentation

### Clinical presentation

**Fig. 2:**
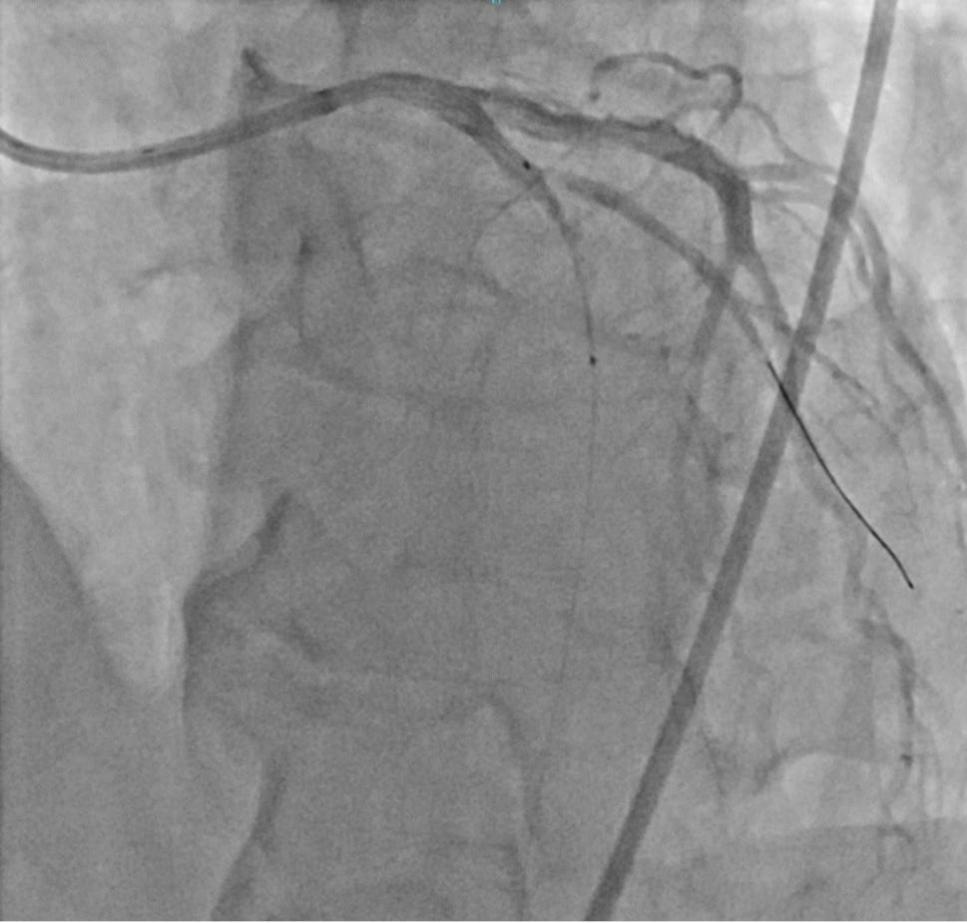
The coronary angiography of patient showed total occlusion in the distal LAD. The revascularization with PCI and thrombus aspiration was not succeed

A 65-year-old male patient was referred to our hospital due to interventricular septal perforation following myocardial infarction. The patient was presented to the previous hospital with chest pain. Despite undergoing percutaneous coronary intervention (PCI) and attempted thrombus aspiration, reperfusion could not be achieved (See Fig. 2). The patient’s condition became unstable, and transthoracic echocardiography (TTE) revealed interventricular septal rupture and left-to-right ventricle shunt. Consequently, the patient was transferred to our hospital. Upon arrival, the electrocardiogram (ECG) showed QS wave on V1-V3 and persistent ST elevation, along with acute kidney injury (AKI) characterized by serum creatinine levels 4–5 times higher than baseline. TTE showed an ejection fraction (EF) of 50%, a mosaic flow near the apical septum, leading to the diagnosis of ventricular septal perforation (VSP), with an akinesia of the anterior left ventricular wall. The patient opted for emergency surgery. The initial procedure involved general anesthesia and cardiopulmonary bypass (CPB) with drainage from the right atrium and a venting tube in the left ventricle (LV). Cardiac arrest was induced through mild hypothermia using antegrade and retrograde cold blood cardioplegia.

### The first surgery

Briefly, a median sternotomy was performed, the pericardium was opened, and the heart was exposed and examined. The right chamber appeared enlarged, and the apex displayed pallor with a wide infarct area. An incision was made in the infarcted region of the LV myocardium, parallel to the interventricular septum, extending approximately 7 cm toward the apex, the VSP then been located (Fig. 1). The double patch technique, as previously described by Takahashi et al. [6], was employed using bovine pericardial patches. The bovine patch was cut with a diameter 2 cm larger than the VSP for the right ventricle (RV) patch (R-patch), while the left ventricle (LV) patch (L-patch) was sized adequately to exclude the VSP, the infarcted area of the LV, and the LV incision. After confirming hemostasis, the heart was closed, and the patient was transferred to the intensive care unit (ICU) (See Fig. 1).

### Follow up

The patient remained in the ICU for 9 days. Dialysis was required initially due to worsening kidney injury from the preoperative AKI, but the patient’s condition stabilized, and continuous dialysis was no longer necessary. TTE performed 2 weeks after surgery indicated a shunt, but no signs of heart failure were observed. Thus, the patient received medication and was discharged 23 days after the surgery.

**Fig. 3:**
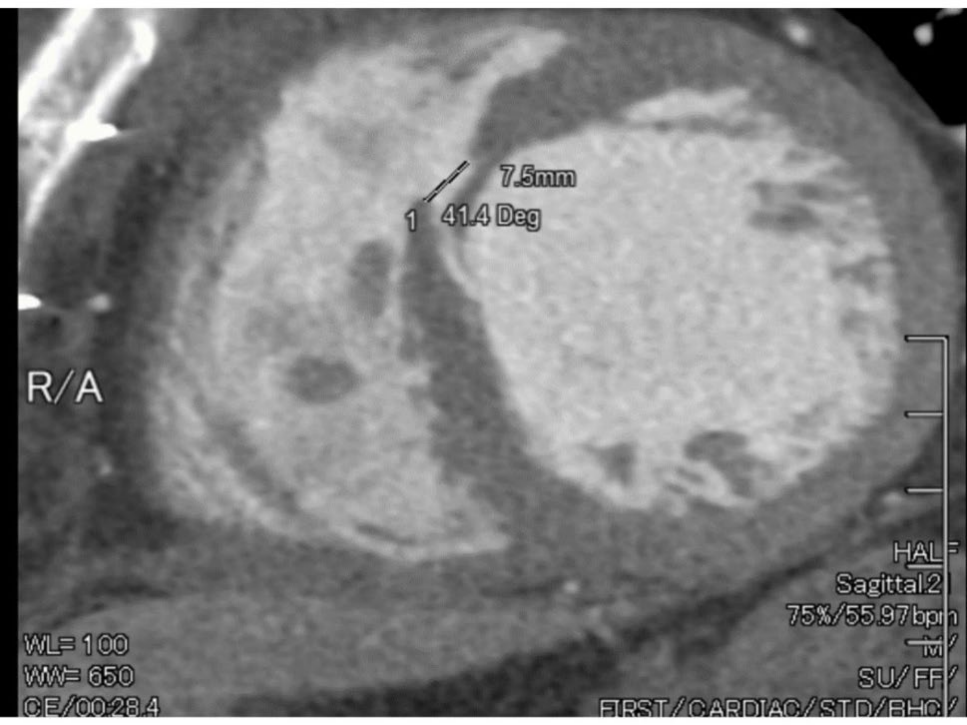
Cardiac CT Scan with contrast before the second surgery indicating VSP

As an outpatient, the patient returned to the hospital after 22 days with chest discomfort. Mild pleural effusion was confirmed, and transesophageal echocardiography (TEE) revealed a left-to-right shunt through the previous patch. Following approximately 6 days of medication administration in the hospital, the patient’s condition stabilized, and discharge was granted. However, the patient returned again after 2 weeks of being discharged, presenting with general malaise and decreased kidney function. TTE showed blood flow from the left to right ventricle, with EF of 55% and Qp/Qs = 1.7. Cardiac computed tomography (CT) and magnetic resonance imaging (MRI) confirmed the presence of a midventricular perforation measuring approximately 12 mm in diameter, leading to the decision to proceed with VSP repair surgery (See Fig. 3).

### The second surgery

**Fig. 4:**
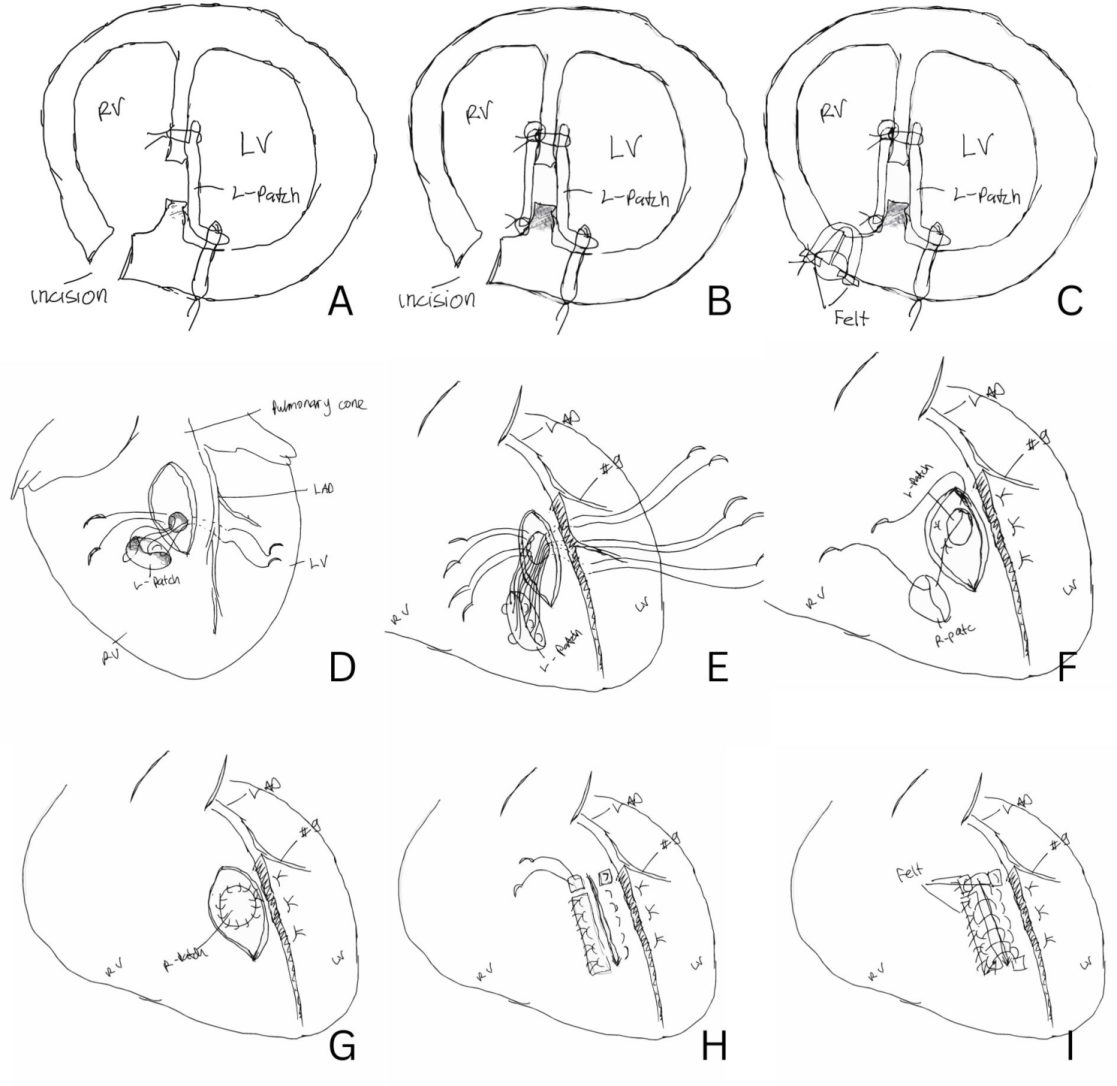
Step by step of the recurrent VSP surgery. (**A**) L-Patch tied in the LV free wall and interventricular septum. (**B**) R-Patch was attached to the interventricular septum and tied with continuous suture. (**C**) RV incision was closed with felt, using mattress suture and double with continuous suture. (**D**) Threads were passed from the LV through the VSD to the LV free wall, and from the LV to the RV through the interventricular septum. (**E**) Five threads were used to attach the L-Patch. (**F**) The L-patch was attached to the LV free wall and interventricular septum, while the R-patch was attached with continuous sutures. (**G**) R-patch already attached. (**H**) RV incision was closed using felt on the edge and right side. (**I**) Another continuous suture was applied

The second surgery was performed on the 15th day after initiating medication in the hospital. A median incision was made, carefully exposing the heart, and establishing a CPB machine. The second surgery employed a double patch technique. Briefly, trans epicardial echocardiogram was performed to locate the VSP. The RV was accessed near the right ventricular outflow tract (RVOT) through a 4 cm incision toward the apex, positioned 1 cm laterally to the left anterior descending artery (LAD). As the distal LAD had already been damaged from the previous myocardial infarction, the RV was cautiously dissected to avoid injury to the first diagonal branch of LAD. The ruptured wall was confirmed, and RVOT was observed, with trabeculae being cut to enhance visibility (Fig. 1). A bovine pericardium patch, measuring 3 cm in diameter and circular in shape, was prepared. The thread was passed from the LV lumen to the LV free wall and from the interventricular septal wall to the RV lumen using 3 − 0 MH sutures. The patch was then pulled into the LV chamber by pulling the thread, and a single ligation was used for suturing, as shown in Fig. 4 Another patch was prepared in a similar manner, with the thread applied from the opposite side and sutured with continuous sutures, securing it to the front side of the interventricular septum. A thread was passed from the LV free wall, near the previous suture, to the RV free wall, closing the RV with teflon felt and tying it with a mattress suture. An additional continuous suture was applied externally between the LV teflon felt and RV teflon felt to ensure closure. A pacing lead was placed in the right atrium and right ventricle, and an intra-aortic balloon pump (IABP) was inserted through the right common femoral artery. After confirming hemostasis, the chest was closed.

The patient remained in the ICU for 5 days, with a maximum creatinine level of 1.55, and experienced no postoperative complications. The IABP was removed 3 days after surgery, and the pacing lead was removed 4 days later. The patient was discharged 4 days after the removal of the pacing lead, displaying stability and no signs of heart failure.

## Discussion and conclusions

Several techniques have been introduced and continue to evolve to treat VSP and prevent leakage, including infarct exclusion, the use of adhesives, and double patch techniques. Less invasive approaches using transcatheter methods have also been developed [5, 7, 8]. VSP following MI remains a challenging surgical procedure that demands a high level of surgical expertise. Surgery in the early phase is considered an option when the patient’s condition deteriorates. The surgery primarily employs either the David technique, which involves infarct exclusion, or the Daggett technique, which utilizes a single or double patch [4]. Several studies have indicated that the use of the infarct exclusion technique with a double patch yields superior outcomes [6, 9, 10]. Recently, transcatheter approaches using a VSP occluder have gained popularity due to their less invasive nature. However, this VSP occluder is not commersially available in Japan, and if a patient is unable to undergo catheterization, open surgery becomes necessary [11]. Various methods of heart incision, such as through the left ventricle (LV), right ventricle (RV), or right atrium, provide better options to maximize results based on the specific location and condition of the VSP in each patient.

The first repair surgery was performed during the early phase of MI, soon after the diagnosis of VSP was established. The decision for emergency surgery was made to enhance the chances of survival, despite the patient’s already poor preoperative condition. The patient had previously undergone unsuccessful PCI, and the distal LAD was completely occluded. During the early phase of MI, distinguishing between necrotic tissue, ischemic areas, and viable tissue was challenging. The initial surgery employed the infarct exclusion double patch technique, with an incision made in the infarcted area through the left ventricle. This approach minimized manipulation of healthy tissue in the right ventricle and preserved the geometry of the left ventricle by avoiding dissection of necrotic tissue. The patient experienced worsening kidney injury after surgery, necessitating dialysis during the hospital stay. However, the patient was later discharged without the need for continued dialysis.

The patient developed a recurrent VSP two weeks after the initial surgery, which was confirmed through echocardiography and CT scan. The previous patch remained intact, and the new VSP appeared in the middle-upper part of the interventricular wall, whereas the initial VSP was located in the apical portion. This complication continues to pose a challenge in the early-phase treatment of VSPs following MI. However, the patient remained stable with medication. Two months later, the patient’s condition worsened, manifesting symptoms of heart failure, leading to the decision for a second surgery. The second surgery employed the same technique but with a different approach, accessing the VSP from the right ventricle (RV). This decision was based on the location of the new VSP in the middle-upper part of the interventricular wall, with the previous patch still intact. Additionally, the left ventricle (LV) already displayed scars from the previous infarction, necessitating caution to avoid further injury. After confirming the location of the VSD through trans-epicardium echocardiography, an incision was made 1 cm laterally to the left anterior descending artery (LAD). The incision was performed meticulously, considering the need to avoid damaging the papillary muscle. Due to occlusion of the distal LAD from the previous MI and the desire to preserve the first diagonal branch of the LAD, the incision was made near the occluded distal LAD. The VSP was relatively small, measuring approximately 10 mm, so instead of attaching both patches with a single suture, each patch was sutured individually. The L-patch was sutured to the LV free wall and interventricular septum using mattress sutures, while the R-patch was attached to the interventricular septum using continuous sutures. In the case of the L-patch, five sutures were used, with three attached to the LV free wall and two attached to the interventricular septum. Despite encountering difficulties due to adhesions from the previous surgery, the operation was successfully performed. The patient remained in the ICU for 5 days and was later discharged without experiencing any symptoms of heart failure.

Repairing a VSP after MI in the early phase presents challenges due to the unclear border between healthy and injured tissue. A systematic review by Perez-Villa et al. stated that using temporary and durable Mechanical Circulatory Support (MCS) as a bridge to heart transplantation (HT) may present a viable approach, either as a primary strategy or in cases of emergency, to lower the elevated in-hospital mortality among patients with MI-related VSP [12]. Despite the various techniques introduced, there is no definitive best technique for repairing recurrent VSPs. Mastery of both RV and LV approaches provides better options based on the location of the VSP in each patient. Recently, although the Amplatzer VSP occluder is not commercially available in Japan and guidelines for treating VSP with other types of Amplatzer devices have not been established, successful reports of percutaneous repair using Amplatzer Septal Occluder (ASO) and Amplatzer Ductal Occluder (ADO) have emerged for high-risk surgical patients with VSPs [11, 13].

## Data Availability

There are no additional data to disclose.
